# Public attitudes challenge clinical practice on genetic risk disclosure in favour of healthcare-provided direct dissemination to relatives

**DOI:** 10.1038/s41431-023-01428-3

**Published:** 2023-07-20

**Authors:** Anna Rosén, Mateja Krajc, Hans Ehrencrona, Svetlana Bajalica-Lagercrantz

**Affiliations:** 1 Department of Radiation Sciences, Oncology, Umeå University, Umeå, Sweden; 2 Department of Clinical Cancer Genetics, Institute of Oncology Ljubljana, Ljubljana, Slovenia; 3 Division of Clinical Genetics, Department of Laboratory Medicine, Lund University, Lund, Sweden; 4 Department of Genetics, Pathology and Molecular Diagnostics, Office for Medical Services, Region Skåne, Lund, Sweden; 5 Department of Oncology-Pathology, Karolinska Institutet, Stockholm, Sweden; 6 Hereditary Cancer Unit, Karolinska University Hospital, Stockholm, Sweden

**Keywords:** Risk factors, Cancer genetics, Cancer prevention, Genetic testing, Disease genetics

## Enabling preventive measures in the era of precision medicine

The increased usage of genetic testing for treatment stratification within the era of precision medicine entails the potential to detect germline genetic risk variants. Germline genetic testing often has implications not only for the individual patient but also for their genetic relatives. This is especially true for high-penetrance pathogenic variants associated with conditions such as familial hypercholesterolemia and hereditary cancer risk syndromes like Lynch syndrome and the hereditary breast and ovarian cancer syndrome. For these conditions, targeted prevention programs are available, and cascade screening is cost-effective [1, 2]. It is therefore highly relevant to find effective strategies to disclose information from the genetic investigation to healthy relatives at risk. Informing relatives at risk enables equitable access to pre-test genetic counselling and a possibility for them to make an informed decision about predictive genetic testing as well as prevention.

## Genetic risk disclosure—time to change to a healthcare-mediated direct approach?

Current practice in most countries is to encourage index patients to inform their relatives about the potential impact of a genetic risk assessment. Several studies have explored the barriers and facilitators of family communication, and some have also tested interventions to improve efficacy. It seems that tailored genetic counselling with additional follow-up can increase both the proportion of informed relatives and relatives who contact the genetics clinic, but the data are not conclusive [3, 4]. However, another large meta-analysis on hereditary cancer risk disclosure shows that with family-mediated disclosure, the uptake of genetic counselling in relatives is about 35%, whereas the uptake almost doubles (63%) when using a healthcare-mediated direct contact approach [5].

## What do people think of direct contact (from a hypothetical point of view)?

In this issue of *European Journal of Human Genetics*, Tiller et al. [6] present interesting data on questionnaires directed to the general public. Hence, most participants lacked the experience of belonging to a family with a disease pattern often seen in a hereditary condition. The respondents were briefly introduced to the concept of medically actionable genetic conditions, the importance of sharing information with (genetic) relatives, and current standard practice with family-mediated risk disclosure. On a hypothetical question, most respondents (85%) expressed a preference for being informed about potential genetic risks for future health problems that can be prevented or treated early. However, it remains unclear to what extent respondents with real-life experience of familial disease aggregation would be in favour of such risk awareness.

When provided with two different types of information letters, 67% of respondents preferred a letter with more specific information about the variant in the family, health risks, and preventive measures, whereas 21% preferred a letter containing more general information. Notably, when asked ***from whom*** they preferred to receive the letter, less than a tenth (8.4%) preferred to receive the letter from a family member. Thus, only a minority preferred to receive information in the way commonly used in current clinical practice in most countries, where the index often is provided with a ‘family letter’ to further distribution to relatives.

Tiller et al. also show that the majority (68%) would prefer that healthcare providers disseminate the letter directly to them. Interestingly, one-third of them would also like to be contacted by a family member for an explanation. The preference that healthcare providers are directly involved in genetic information disclosure to at-risk relatives is in agreement with reports of public opinion data from Belgium [7], Sweden [8], and Denmark [9]. The Australian data contribute to an increasing body of evidence showing that—in a hypothetical situation—the public envisions a practice that is not implemented today.

## Future perspectives

Even though family-mediated disclosure is well established, it entails complex ethical questions. For example, the dissemination of information to at-risk relatives depends on the willingness of the index case. An approach with direct healthcare-provided risk disclosure could also be challenging. There are only a limited number of studies investigating the real experience of receiving risk information through healthcare-provided direct contact. Even though these studies indicate that the direct approach may be accepted [9] and safe concerning anxiety levels [10, 11], further analyses of the impact on at-risk individuals are needed. There is also a lack of studies evaluating the effectiveness of the direct contact approach concerning the uptake of cascade testing among family members and the degree of enrolment in surveillance. This could preferably be approached by large randomised controlled trials. Of note, the implementation of the direct approach challenges issues concerning patient’s autonomy and confidentiality, and relatives right not to know. On the other hand, the direct approach has the potential to safeguard the relatives’ right to receive information with potential relevance for their health, i.e., their right to know.

The healthcare system faces a growing need to accommodate an ever-increasing number of (healthy) at-risk individuals. Supplementing family-mediated disclosure with healthcare-provided direct information could constitute an improvement. Hereditary aspects of germline genetic findings are challenging healthcare to a paradigm shift from the patient to the family as the unit of care. This involves a multitude of aspects including not only how to practically identify and define at-risk individuals but also how to administer and store family-level data. Country-specific legislation and privacy rules govern the possibilities and limitations of these endeavours.
